# Genetic characterization of upper respiratory tract virome from nonvaccinated Egyptian cow-calf operations

**DOI:** 10.1371/journal.pone.0267036

**Published:** 2022-05-05

**Authors:** Abdou Nagy, Fatma Abdallah, Hend M. El Damaty, Ahmed Tariq, Abdallah M. A. Merwad, Bader Y. Alhatlani, Ibrahim Elsohaby

**Affiliations:** 1 Department of Virology, Faculty of Veterinary Medicine, Zagazig University, Zagazig City, Sharkia, Egypt; 2 Infectious Diseases, Department of Animal Medicine, Faculty of Veterinary Medicine, Zagazig University, Zagazig City, Sharkia, Egypt; 3 Veterinarian, Zagazig City, Sharkia, Egypt; 4 Department of Zoonoses, Faculty of Veterinary Medicine, Zagazig University, Zagazig City, Sharkia, Egypt; 5 Department of Applied Medical Sciences, Applied College, Qassim University, Unayzah, Saudi Arabia; 6 Department of Health Management, Atlantic Veterinary College, University of Prince Edward Island, Charlottetown, Prince Edward Island, Canada; 7 Department of Infectious Diseases and Public Health, Jockey Club of Veterinary Medicine and Life Sciences, City University of Hong Kong, Kowloon, Hong Kong; Taif University, SAUDI ARABIA

## Abstract

Bovine respiratory disease (BRD) is the costliest complex disease affecting the cattle industry worldwide, with significant economic losses. BRD pathogenesis involves several interactions between microorganisms, such as bacteria and viruses, and management factors. The present study aimed to characterize the nasal virome from 43 pooled nasal swab samples collected from Egyptian nonvaccinated cow-calf operations with acute BRD from January to February 2020 using metagenomic sequencing. Bovine herpesvirus-1 (BHV-1), first detection of bovine herpesvirus-5 (BHV-5), and first detection of bovine parvovirus-3 (BPV-3) were the most commonly identified in Egyptian cattle. Moreover, phylogenetic analysis of glycoprotein B revealed that the BHV-1 isolate is closely related to the Cooper reference strain (genotype 1.1), whereas the BHV-5 isolate is closely related to the reference virus GenBank NP_954920.1. In addition, the whole-genome sequence of BPV-3 showed 93.02% nucleotide identity with the reference virus GenBank AF406967.1. In this study, several DNA viruses, such as BHV-1 and first detection BHV-5, and BPV-3, were detected and may have an association with the BRD in Egyptian cattle. Therefore, further research, including investigating more samples from different locations to determine the prevalence of detected viruses and their contributions to BRD in cattle in Egypt, is needed.

## Introduction

Bovine respiratory disease (BRD) is one of the most economically important disease affecting the cattle industry worldwide [1]. The cause of BRD is multifactorial, including infectious agents and environmental, management, and host factors. The infectious agents include different viruses, such as bovine herpesvirus-1 (BHV-1), bovine viral diarrhea virus (BVDV), bovine coronavirus, bovine respiratory syncytial virus (BRSV), parainfluenza 3 virus, bovine adenovirus, influenza D virus (IDV) and bovine rhinitis virus, and bacteria, such as *Mycoplasma bovis*, *Pasteurella multocida*, and *Mannheimia haemolytica* [2]. Despite the widespread use of BRD vaccines and different effective antibiotics, mortalities caused by BRD have steadily increased since the mid-1990s [3, 4]. This indicates that the etiology and pathogenesis of BRD are not fully understood.

Recently, viral metagenomics has been used to characterize the virome associated with complex diseases [5–8]. Viral metagenomics has been used to identify large numbers of known and novel viruses associated with enteric diseases [9, 10] and respiratory diseases [11, 12]. Previous studies on the bovine respiratory tract using metagenomic sequencing showed a significant association between bovine adenovirus-3 (BAdV-3), bovine rhinitis A and B virus, BRSV, BPIV3, BHV-1, BVDV, bovine parvovirus (BPV), IDV, and BRD [7, 11, 13]. BHV-1 and bovine herpesvirus-5 (BHV-5) are DNA viruses that belong to the family *Herpesviridae*. Although BHV-1 causes low mortality, it is responsible for severe economic loss to the cattle industry due to its impact on growth and milk production [14]. BHV-5 causes nonsuppurative meningoencephalitis in young cattle [15].

There are six bovine parvovirus sub-species, and the most significant three sub-species are BPV-1 to BPV-3, which cause diarrhea in neonatal calves and respiratory and reproductive diseases in adult cattle [16]. In addition to these viruses, recent metagenomic studies showed a significant association between IDV and BRD in dairy calves [11] and beef cattle [13].

Egypt has a big cattle industry, with open markets for live-animal trade with different countries, mainly from Europe and Africa. Although the control of certain viruses that may contribute to the complex pathogenesis of BRD, such as BVDV, and infectious bovine rhinotracheitis has been successfully achieved with strict vaccination programs, particularly in large farms, mortality due to BRD has increased [17]. To the authors’ knowledge, no data are available on viruses that contribute to the BRD incidence in cattle in Egypt. Therefore, the purpose of this study was to characterize the virome of cattle with the BRD to identify possible viruses of interest for future investigation in a case-control design.

## Materials and methods

### Ethical approval

This study was approved by the Zagazig University’s Animal Care and Use Committee (Ref. No. ZU-IACUC/2/F/121/2019). The farm owners provided informed verbal/written consent for the use of clinical samples collected from their animals in the present study.

### Study population

A total of 43 nasal swabs were collected from eight cow-calf operations in Sharkia (n = 27) and Cairo (n = 16) Governorates. The average number of cattle per herd was 264. The age of cattle at the time of sampling ranged from 1 to 18 months, and the weight ranged from 40 to 450 kg. There were 81.4% (35/43) males and 18.6% (8/43) females. All samples were collected from nonvaccinated cattle. Nasal swab samples were collected only from diseased animals with severe clinical respiratory signs, such as cough, difficulty breathing, and rhinorrhea.

### Sample collection

Nasal swabs were collected (one per animal) from cattle herds from January to February 2020. All samples were collected from nonvaccinated cattle of various ages, raised in either large intensive and/or small backyard farms. All swabs were collected from naturally infected animals exhibiting severe clinical signs of acute respiratory disease (i.e., cough, difficulty breathing, rhinorrhea, and ocular discharge). Swabs were collected by certified veterinarians using sterile cotton swabs followed by insertion into Falcon tube containing 1 ml viral transport medium composed of sterile phosphate-buffered saline, supplemented with 1% penicillin/streptomycin (10,000 U/ml; Gibco, USA). The collected swabs were labelled with herd and animal ID and the collection date and then shipped on ice to the virology laboratory for processing and testing.

### Sample preparation and DNA extraction

Nasal swabs were vortexed separately and then centrifuged at 2000 rpm for 10 min at 4°C for clarification. The clear supernatant was collected from each processed sample and stored at −20°C until used. Viral nucleic acid was extracted from each clear supernatant using the GeneJET Viral DNA extraction/purification kit (Thermo Fisher, MA, USA) according to the manufacturer’s instructions. The extracted viral DNA was eluted in water-nuclease free. The extracted viral DNAs from all samples (n = 43) were pooled and loaded to the FTA card and sent to the Admera’s Health LLC (South Plainfield, NJ, USA) for library preparation and next-generation sequencing.

### Library preparation and sequencing

Isolated nucleic acid was quantified with Qubit 2.0 DNA HS Assay (Thermo Fisher) and the quality was assessed by Tapestation Genomic DNA Assay (Agilent Technologies, CA, USA). Library preparation was performed using the NexteraXT library kit (Illumina, CA, USA) according to the manufacturer’s recommendations. The final library quantity was measured by KAPA SYBR^®^ FAST qPCR with QuantStudio^®^ 5 System (Applied Biosystems, CA, USA), and library quality was evaluated by TapeStation HSD1000 ScreenTape (Agilent Technologies). Illumina^®^ 8-nt dual indices were used. Equimolar pooling of libraries in the same run was performed based on QC values and sequenced on an Illumina^®^ NovaSeq with a read length configuration of 150 paired-end.

### Bioinformatic analysis

Data were analyzed according to a customized pipeline. In brief, raw FASTQ files were initially checked for quality control using FastQC software version 0.11.8 (https://www.bioinformatics.babraham.ac.uk/projects/fastqc). In brief, quality of reads were assessed for; per base sequence quality, per tile sequence quality, per base sequence content, per base N content, per sequence quality scores, per sequence GC content, levels of sequence duplications, overrepresented sequences, sequence length distribution and adapter content. High-quality files were imported into Geneious Prime 2020 (Biomatters Ltd., New Zealand), and all reads were first trimmed using default settings. In brief, BBDuk was used with the options checked to “Trim Adapters” (all Trueseq, Nextera, and PhiX adapters), trim low quality (minimum quality of 25), discard short reads (minimum length of 40 bp), and keep original order. Duplicates were removed after trimming using sequence -> Remove duplicate reads.

The processed/trimmed reads were then mapped to the host reference genome (Bos taurus, PRJNA33843) and the unmapped reads were subjected to de novo assembly into contigs using Velvet with default settings. Consequences sequences (150–200 bp) were extracted from contigs and analyzed against the nucleotide database of NCBI using BLASTN tool. Viral genomes with very low expectation value (E-value ~ 10^−4^) were used as reference viruses for reads mapping using Geneious Prime mapper default settings with the options Medium Sensitivity/Fast Sensitivity setting that were modified by selecting the Custom Sensitivity, and Fine Tuning was set to iterate up to five times. A consensus sequence was constructed based on the highest quality threshold and extracted using “Tools” -> “Generate consensus sequence,” calling “?” (base or gap) in the absence of coverage. BLAST tool was used to confirm the specificity of the reads aligned to each reference virus using the Megablast algorithm against the nonredundant nucleotide database of GenBank, EMBL, DDBJ, PDB, and RefSeq. The matching region of the best hit per read was retrieved. All original fastaQC files were submitted to the Sequence Read Archive (SRA) database of the National Center for Biotechnology Information (NCBI). Open Reading Frame (ORF) finder algorithm embedded in Geneious Prime was used to detect ORFs from the identified viruses.

### Phylogenetic analysis

Nucleotide sequences from genetically related viruses for BHV-1, BHV-5 and BPV-3 were downloaded from the NCBI database and aligned separately using MUSCLE [18] embedded in MEGA X with the default settings [19]. The best-fitted model of evolution was identified for each alignment using the Bayesian Information Criterion in MEGA X, and neighbor-joining trees with model/method “Maximum Composite Likelihood” were reconstructed using 1000 bootstrap replicates to evaluate the strength of branching [19].

## Results

### Samples collection and processing

**Fig 1:**
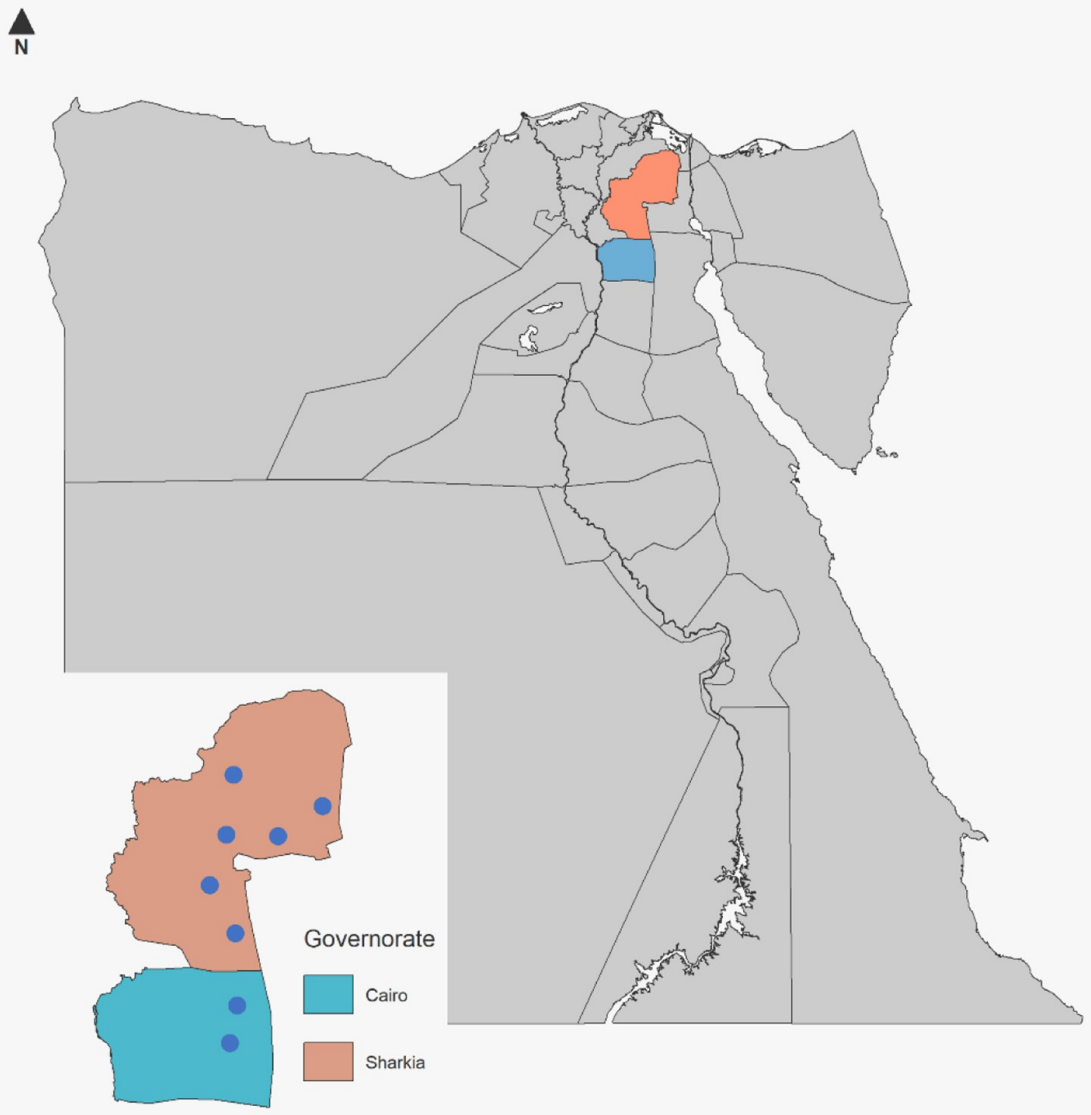
A map of herd locations in Egypt from which animal’s samples were collected.

Samples were collected from different cattle herds located at Cairo and Sharkia Governorates. Details of the locations of the herds where samples were collected are shown in Fig 1. Additionally, details about diseased animals, such as age, breed, type of the ration provided, the antimicrobials used, type of the farm rearing system are presented in (S1 Table). All samples were processed and the viral DNA was extracted (30 μl/sample), pooled (10 μl/sample). A volume of 100 μl from the final pooled viral DNAs was uploaded to one FTA card that was shipped for library preparation and next-generation sequencing.

### Virome of bovine nasal swabs

A total of 56 million raw reads generated by the sequencing machine were submitted to the SRA under the Bioproject number PRJNA702539. Following de novo assembly, six DNA viruses were identified (Table 1). For the best blast hits, reference genomes of the identified viruses were downloaded from GenBank database and the final processed/trimmed reads (n = 8 millions) were mapped against them. All alignments were inspected manually with a threshold of minimum 20 processed/trimmed reads mapped to different regions of the reference viral genome was the virus considered detected.

**Table 1:** Summary of the identified DNA viruses.

Reference virus ^1^	Family	GenBank accession number ^2^	Genome size (bp)	No. positive reads	Largest contig size (bp)	Further analysis ^3^
BHV-1	*Herpesviridae*	AJ004801.1	135,301	5958	2523	Yes
BHV-5	*Herpesviridae*	KY559403.1	137,740	4523	2661	Yes
MdSGHV	*Hytrosaviridae*	EU522111.1	124,279	1686	71	No
BPV-3	*Parvoviridae*	AF406967.1	5276	593	1283	Yes
BAdV-3	*Adenoviridae*	AF030154.1	34,446	26	46	No

^1^ BHV-1: Bovine herpesvirus 1; BHV-5: Bovine herpesvirus 5; MdSGHV: Musca domestica salivary gland hypertrophy virus; BPV-3: Bovine parvovirus 3; BAdV-3: Bovine adenovirus

^2^ GenBank numbers are for reference virus genomes used for processed/trimmed reads mapping.

^3^ Samples with high coverage (~98%) and long contigs against the reference virus genomes were further investigated for annotation and phylogenetic analysis.

Additionally, other viruses such as bovine adeno-associated virus, bovine torovirus, bovine nidovirus, and bovine hokovirus 2 were identified. However, due to the small number of reads (<20) mapped against these viruses and short length of contigs, they were considered as false-positive and so, they were excluded from further analysis.

### Bovine herpesviruses

**Fig 2:**
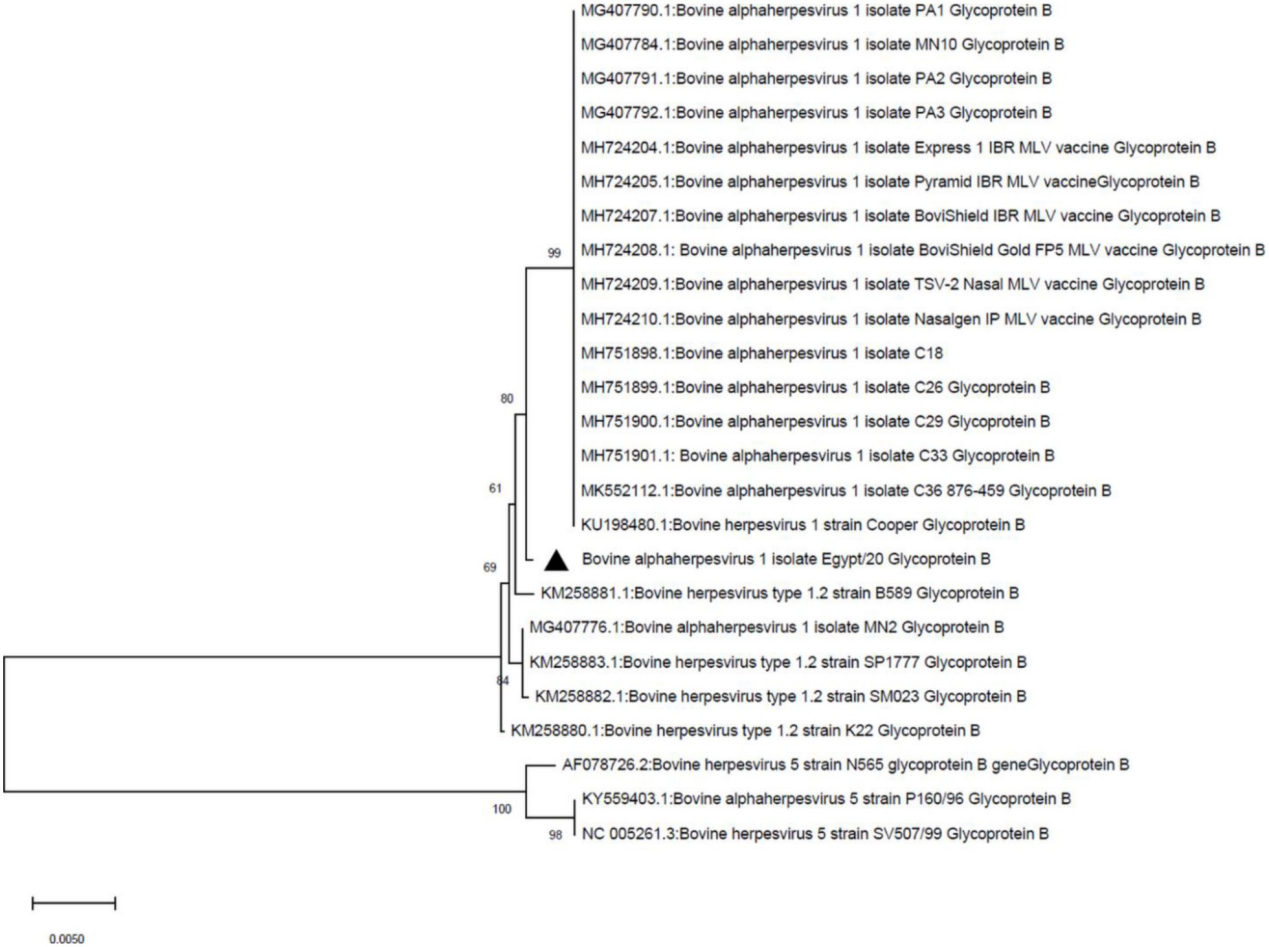
Phylogenetic analysis of BHV-1 gB. The evolutionary history was inferred using the neighbor-joining method [20]. The percentages of the replicate trees in which the associated taxa clustered together in the bootstrap test (1000 replicates) are shown next to the branches [21]. The evolutionary distances were computed using the maximum composite likelihood method [22] and are shown as the number of base substitutions per site. The codon positions included were 1^st^ + 2^nd^ + 3^rd^ + Noncoding. Evolutionary analyses were conducted in MEGA X [19]. Different identical sequences were included to increase the robustness of the constructed tree.

**Fig 3:**
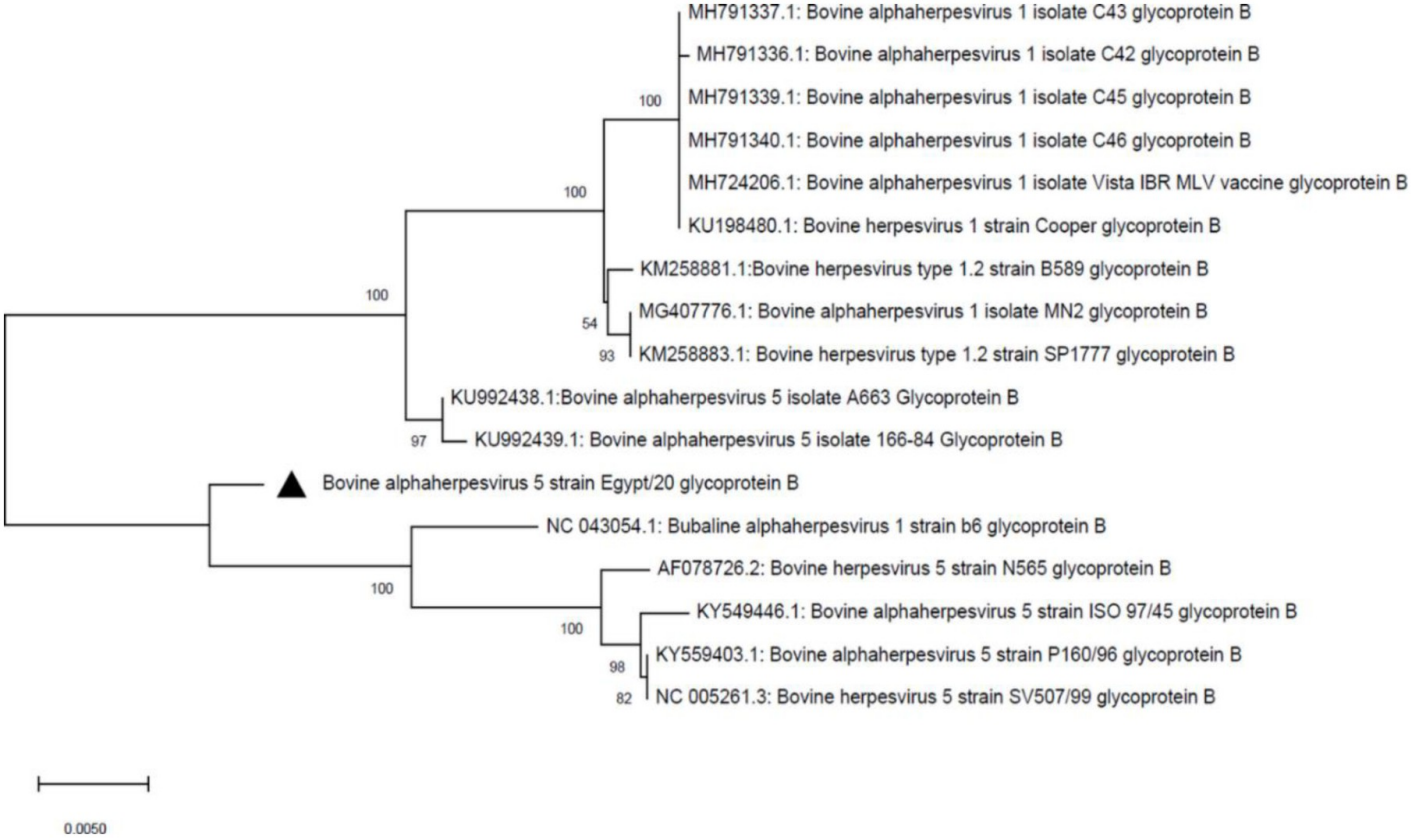
Phylogenetic analysis of BHV-5 gB. The evolutionary history was inferred using the neighbor-joining method [20]. The percentages of replicate trees in which the associated taxa clustered together in the bootstrap test (1000 replicates) are shown next to the branches [21]. The evolutionary distances were computed using the maximum composite likelihood method [22] and are shown as the number of base substitutions per site. The codon positions included were 1^st^ + 2^nd^ + 3^rd^ + Noncoding. Evolutionary analyses were conducted in MEGA X [19]. Different identical sequences were included to increase the robustness of the constructed tree.

A total of 5958 and 4523 processed/trimmed reads were mapped to the reference viruses; BHV-1 (GenBank AJ004801.1) and BHV-5 (GenBank KY559403.1), respectively. For BHV-1, analysis of the viral glycoproteins, such as glycoprotein B (gB), showed 99.57% nucleotide identity with other BHV-1 viruses, such as the Cooper reference strain, which was isolated from USA. In addition, the study isolate (called BHV-1/Cattle/Egypt/2020) was clustered with other BHV-1 viruses, such as the Cooper reference strain, BHV-1 isolate C18, C26, C29, C33 and C36 that have been collected from the USA (Fig 2). Moreover, 20 different genes from BHV-1/Cattle/Egypt/2020isolate showed high coverage (98–99%) when mapped to the reference BHV-1 viruses. The 20 characterized genes from BHV-1/Cattle/Egypt/2020 isolate were deposited to the NCBI database (GenBank MW805254-MW805273). On the other hand, analysis of gB from the BHV-5 isolate (called BHV-5/Cattle/Egypt/2020; GenBank MW805274) showed 98.28% nucleotide identity with reference BHV-5 viruses (GenBank KU KY559403.1 and NC_005261.3) and was clustered with the BHV-5 isolate 166–84 and BHV-5 isolate A663 that have been isolated from Argentina (Fig 3).

### BPV-3

**Fig 4:**
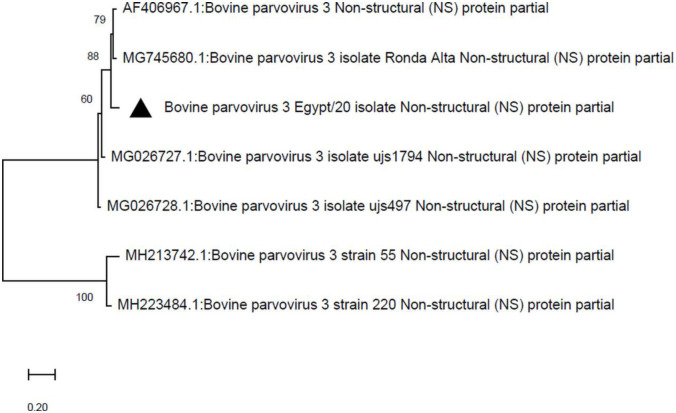
Phylogenetic analysis of the partial NS protein gene of BPV-3. The evolutionary history was inferred using the neighbor-joining method [20]. The percentages of replicate trees in which the associated taxa clustered together in the bootstrap test (1000 replicates) are shown next to the branches [21]. The evolutionary distances were computed using the maximum composite likelihood method [22] and are shown as the number of base substitutions per site. The codon positions included were 1^st^ + 2^nd^ + 3^rd^ + Noncoding. Evolutionary analyses were conducted in MEGA X [19].

A total of 593 processed/trimmed reads were mapped against the reference BPV-3 virus (GenBank AF406967.1). The alignment of the whole-genome sequence of the study isolate (called BPV-3/Cattle/Egypt/2020) showed 93.02% nucleotide identity with the BPV-3 reference strain (GenBank AF406967.1), which was isolated from the USA and 91.75% nucleotide identity with the Ronda Alta isolate (GenBank MG745680.1), which was isolated from Brazil. Additionally, two main open reading frames (ORFs) were detected: (1) ORF1 is the (NS) protein starting from the nucleotide position 264 to 2222 with 97.24% amino acid identity with the reference BPV-3 virus (GenBank AAL09673.1) and (2) ORF2 is the capsid protein starting from the nucleotide position 2171 to 5068 with 97.89% amino acid identity with the reference BPV-3 virus (GenBank AAL09674.1). Moreover, phylogenetic analysis of the partial non-structural (NS) protein gene showed that BPV-3/Cattle/Egypt/2020 clustered with BPV-3 viruses (GenBank MG745680.1, isolated from Brazil), MG026727, and MG026728 (both have been isolated from China); (Fig 4). The whole-genome sequence of the study isolate (called BPV-3/Cattle/Egypt/2020) was deposited to the NCBI database (GenBank MW805276).

## Discussion

BRD is one of the most economically important complex disease affecting the cattle industry worldwide. Despite the use of several vaccines, BRD still has a worldwide distribution, with severe economic losses every year. Egypt has a big cattle industry and live-animal trade with different countries in Europe and Africa, which may facilitate the incidental introduction of new agents. The cattle industry in Egypt is based on two systems: (1) large farms where the average number of animals/herd is 100 to 1000 and (2) small farms where the average number of animals/herd is 5 to 20. Unfortunately, a high percentage of small farms do not apply annual BRD vaccination programs. Moreover, large farms are also not vaccinating young calves (average age of 6–12 months) against BRD.

In the present study, we investigated the nasal virome from naturally infected nonvaccinated cattle using metagenomic sequencing. We aimed to investigate contribution of viruses, particularly DNA viruses to the BRD in Egyptian cattle. Interestingly, several DNA viruses were identified in this study. Deep analysis of the BHV-1/Cattle/Egypt/2020 isolate revealed it belongs to genotype BHV-1.1, which is in agreement with previous studies [23, 24] that reported BHV-1.1 is the major circulating genotype in Egypt. Additionally, analysis of other genes from BHV-1/Cattle/Egypt/2020, such as glycoproteins C and L and the major capsid protein, did not show a significant number of mutations when aligned with reference viruses (data not shown).

Alignment of the processed reads also showed high coverage against BHV-5 reference virus. BHV-5 is a viral cattle disease responsible for causing sporadic epizootics of fatal meningoencephalitis [25, 26]. BHV-5 is similar to BHV-1 in virion morphology, induced cytopathic effect on infected cell cultures, and antigenic properties [25, 27]. BHV-5 is formerly considered a neuropathogenic variant of BHV-1. However, many subsequent studies indicated that both viruses have different antigenic and genomic characters. BHV-1 and BHV-5 have neurotropic forms, but only BHV-5 can replicate well in the central nervous system causing neurological diseases [28, 29]. Meningoencephalitis outbreaks caused by BHV-5 have been reported in many countries, such as Australia [25], North and South America [30–32], and Europe [33, 34]. In addition, the natural transmission of BHV-5 via contaminated semen has been reported in Australia [35]. The virus has also been isolated from cryopreserved semen collected from a healthy bull [36]. Moreover, another study reported that using two species-specific nested PCR that differentiated BHV-1 and BHV-5, BHV-5 DNA was detected in all semen samples analyzed [37], while BHV-1 was detected only in 44.7% of tested samples. Surprisingly, BHV-5 DNA has been identified before in the central nervous system of the aborted fetus that gave an indication of the association of BHV-5 with bovine abortion [38]. It was surprising to detect BHV-5 sequence from nasal swab sample as this virus has high tropism for nervous and genital systems. However, BHV-5 is usually establish latent infection in nervous ganglion, and it has been shown that under stress conditions and/or excessive glucocorticoids treatments, the virus reactivates. During this reactivation stage, BHV-5 can be excreted in nasal, ocular and genital discharges [39].

To our knowledge, this is the first detection of BHV-5 in Egypt. In the present study, it is unclear the source of BHV-5 introduction to Egypt; however, it is critical to carefully examine the imported frozen semen batches at artificial insemination centers using molecular biology techniques to avoid widespread of BHV-5 and to minimize its drawbacks on the cattle industry in Egypt.

The third virus detected was BPV-3/Cattle/Egypt/2020 with a whole-genome sequence of ~5286 nt long. Bovine parvovirus (BPV) belongs to genus Bocaparvovirus genus in the family *Parvoviridae* and was first discovered in 1961 from the gastrointestinal tract of diarrheal calves [40]. BPV has also been associated with reproductive disorders, such as spontaneous abortions and stillbirths [41]. To our knowledge, this is also the first detection of BPV type 3 in Egyptian cattle herds.

This work demonstrates the utility of metagenomic sequencing for the effective detection of viruses in cattle. There are some limitations in the present study, particularly samples pooling and the use of FTA cards. It has been reported that pooling of DNA generated challenges for accurate variant call and allele frequency. For example, sequencing errors confound with the alleles present at low frequency in the pools probably give rise to false-positive variants [42]. In addition, it has been reported that next-generation sequencing of nucleic acid samples derived from FTA cards exhibited lower proportions of poliovirus specific reads with a lower percentage of genome mapped than those obtained directly from viral isolates [43].

## Conclusions

The current study shows the detection of BHV-1.1 genotype and the first detection of BHV-5 and BPV-3 from the investigated cattle herds. The detected viruses may have an association with the BRD; however, further research, including collecting and investigating more animal samples from different locations, is needed to determine the prevalence of the detected viruses and their contributions to BRD in cattle in Egypt.

## Supporting information

S1 Table Details about investigated cattle herds. (DOCX)
