# Supplementary material for: Genetic characterization of upper respiratory tract virome from nonvaccinated Egyptian cow-calf operations
Source: PLoS One. 2022 May 5;17(5):e0267036. doi: 10.1371/journal.pone.0267036 (PMC9070947; doi:10.1371/journal.pone.0267036)
Supplement: S1 Table — (DOCX) [file pone.0267036.s001.docx]

**S1 Table.** Details about investigated cattle herds.

| **Sample code** | **Sex** | **Age (months)** | **Weight (kg)** | **Antimicrobial**  **Medications*** | **No. of heads** | **Floor type** | **Province** |
| --- | --- | --- | --- | --- | --- | --- | --- |
| CaC1 | Male | 8 | 120 | Draxxin | ND** | Concrete | Sharkia |
| CaC2 | Male | 8 | 150 | Nuflor | 600 | Sand | Cairo |
| CaC3 | Male | 8 | 150 | Nuflor | 600 | Sand | Cairo |
| CaC4 | Male | 11 | 200 | Nuflor | 600 | Sand | Cairo |
| CaC5 | Male | 9 | 180 | Nuflor | 600 | Sand | Cairo |
| CaC6 | Male | 10 | 200 | Nuflor | 600 | Sand | Cairo |
| CaC7 | Male | 10 | 200 | Nuflor | 600 | Sand | Cairo |
| CaC8 | Male | 10 | 200 | Nuflor | 600 | Sand | Cairo |
| CaC9 | Male | 7-8 | 150 | Nuflor | 600 | Sand | Cairo |
| CaC10 | Male | 8 | 150 | Nuflor | 600 | Sand | Cairo |
| CaC11 | Male | 7 | 120 | Nuflor | 600 | Sand | Cairo |
| CaC12 | Male | 12 | 250 | Nuflor | 600 | Sand | Cairo |
| CaC13 | Male | 7 | 120 | Nuflor | 600 | Sand | Cairo |
| CaC14 | Male | 14 | 300 | Nuflor | 600 | Sand | Cairo |
| CaC15 | Male | 12 | 200 | Nuflor | 600 | Sand | Cairo |
| CaC16 | Male | 7 | 100 | Nuflor | 600 | Sand | Cairo |
| CaC17 | Male | 8 | 160 | Nuflor | 600 | Sand | Cairo |
| CaC18 | Male | 10 | 200 | Draxxin | 3 | Concrete | Sharkia |
| CaC19 | Male | 3 | 80 | Draxxin | 50 | Soil | Sharkia |
| CaC20 | Male | 2 | 60 | Draxxin | 50 | Soil | Sharkia |
| CaC21 | Male | 3 | 60 | Draxxin | 50 | Soil | Sharkia |
| CaC22 | Male | 3 | 50 | Draxxin | 50 | Soil | Sharkia |
| CaC23 | Female | 3 | 80 | Draxxin | 50 | Soil | Sharkia |
| CaC24 | Male | 3 | 60 | Draxxin | 50 | Soil | Sharkia |
| CaC25 | Male | 3 | 70 | Draxxin | 50 | Soil | Sharkia |
| CaC26 | Male | 13 | 300 | Draxxin | 50 | Soil | Sharkia |
| CaC27 | Male | 4 | 80 | Draxxin | 50 | Soil | Sharkia |
| CaC28 | Female | 4 | 90 | Draxxin | 50 | Soil | Sharkia |
| CaC29 | Male | 5 | 120 | Draxxin | 50 | Soil | Sharkia |
| CaC30 | Male | 5 | 100 | Draxxin | 50 | Soil | Sharkia |
| CaC31 | Male | 1 | 40 | Draxxin | 50 | Soil | Sharkia |
| CaC32 | Female | 18 | 250 | Draxxin | 2 | Concrete | Sharkia |
| CaC33 | Female | 6 | 110 | Draxxin | 5 | Concrete | Sharkia |
| CaC34 | Female | 4 | 70 | Draxxin | 150 | Soil | Sharkia |
| CaC35 | Female | 4 | 70 | Draxxin | 150 | Soil | Sharkia |
| CaC36 | Male | 4 | 65 | Draxxin | 150 | Soil | Sharkia |
| CaC37 | Female | 18 | 300 | Tylovet | 150 | Soil | Sharkia |
| CaC38 | Female | 5 | 450 | Tylovet | 150 | Soil | Sharkia |
| CaC39 | Male | 14 | 300 | Draxxin | 5 | Concrete | Sharkia |
| CaC40 | Male | 8 | 130 | Draxxin | 15 | Concrete | Sharkia |
| CaC41 | Male | 7 | 100 | Draxxin | 15 | Concrete | Sharkia |
| CaC42 | Male | 6 | 100 | Draxxin | 15 | Concrete | Sharkia |
| CaC43 | Male | 7 | 110 | Draxxin | 15 | Concrete | Sharkia |

* Antimicrobials used in the study herds are mainly;

- Draxxin contains Tulathromycin as an active principle and is manufactured by Zoetis company.
- Nuflor contains Florfenicol as the active principle and is manufactured by MSD animal health company.
- Tylovet 20% contains Tylosin as the active principle and is manufactured by PharmaSwede company.

** ND: Not determined.

**Notes:**

- Breed: all cattle involved in the study are hybrid breeds resulting from breeding between native Egyptian cow and foreign (Western) breeds such as Holstein.
- Diet: all cattle involved in the study are fed on a concentrated diet that consists of corn, soya bean seeds, cotton seeds without oil and wheat bran.
